# Pharmacological Characterization of [^18^F]-FNM and Evaluation of NMDA Receptors Activation in a Rat Brain Injury Model

**DOI:** 10.1007/s11307-023-01811-y

**Published:** 2023-03-21

**Authors:** Marie Beaurain, Franck Talmont, Damien Pierre, Patrice Péran, Samuel Boucher, Anne Hitzel, Marie-Pierre Rols, Olivier Cuvillier, Pierre Payoux, Anne-Sophie Salabert

**Affiliations:** 1 Toulouse NeuroImaging Center, ToNIC, UMR1214 Inserm, Toulouse, France; 2 Nuclear Medicine Department, Toulouse Purpan University Hospital, Toulouse, France; 3 Institut de Pharmacologie Et de Biologie Structurale, Université de Toulouse, CNRS, UPS, 31059 Toulouse, France

**Keywords:** Neuroimaging, NMDA receptors, Positron-emission tomography

## Abstract

**Purpose:**

NMDA receptors (NMDARs) dysfunction plays a central role in the physiopathology of psychiatric and neurodegenerative disorders whose mechanisms are still poorly understood. The development of a PET (positron emission tomography) tracer able to selectively bind to the NMDARs intra-channel PCP site may make it possible to visualize NMDARs in an open and active state. We describe the *in vitro* pharmacological characterization of [^18^F]-fluoroethylnormemantine ([^18^F]-FNM) and evaluate its ability to localize activated NMDA receptors in a rat preclinical model of excitotoxicity.

**Procedures:**

The affinity of the non-radioactive analog for the intra-channel PCP site was determined in a radioligand competition assay using [^3^H]TCP ([^3^H]N-(1-[thienyl]cyclohexyl)piperidine) on rat brain homogenates. Selectivity was also investigated by the displacement of specific radioligands targeting various cerebral receptors. *In vivo* brain lesions were performed using stereotaxic quinolinic acid (QA) injections in the left motor area (M1) of seven Sprague Dawley rats. Each rat was imaged with a microPET/CT camera, 40 min after receiving a dose of 30 MBq + / − 20 of [^18^F]-FNM, 24 and 72 h after injury. Nine non-injured rats were also imaged using the same protocol.

**Results:**

FNM displayed IC_50_ value of 13.0 ± 8.9 µM in rat forebrain homogenates but also showed significant bindings on opioid receptors. In the frontal and left somatosensory areas, [^18^F]FNM PET detected a mean of 37% and 41% increase in [^18^F]FNM uptake (*p* < 0,0001) 24 and 72 h after QA stereotaxic injection, respectively, compared to the control group.

**Conclusions:**

In spite of FNM’s poor affinity for NMDAR PCP site, this study supports the ability of this tracer to track massive activation of NMDARs in neurological diseases.

## Introduction   

N-methyl-D-aspartate receptors (NMDARs) are glutamate ionotropic receptors that regulate excitatory synaptic transmission and are widely expressed in the central nervous system (CNS). They play an essential role in many biological functions, including neurotransmission, neuroprotection, neurodegeneration, long-term potentiation, memory, and neurogenesis [1]. However, NMDARs also contribute importantly to the etiology and progression of many neurological disorders. Detrimental effects can result from either hyperactivity of NMDARs, leading to excitotoxic cell death as in stroke and brain trauma [2], epileptogenesis [3], neurodegenerative diseases [4], or hypofunction, as likely occurs in schizophrenia [5].

NMDARs are heterotetramers made up of three different families of subunits: GluN1, GluN2, and GluN3. The ion channel is formed by two necessary GluN1 subunits and either two GluN2 subunits or a combination of GluN2 and GluN3 subunits. The activation of NMDARs requires two different processes. First, the simultaneous binding of the co-agonists L-glutamate and L-glycine (or D-serine) to their own binding extracellular site. These sites are located on the GluN2 subunit for the glutamate, and on the GluN2 and GluN3 subunits for the glycine. Second, the expulsion of the Mg^2+^ cation blocking the intra-channel site by membrane depolarization, thus allowing the ion flux through the channel. Under physiological conditions, NMDARs are opened for only brief period of time and mediate long-term potentiation by allowing the influx of Ca^2+^ ions as well as Na^+^ and K^+^ into the synapse. However, excessive glutamate release following cellular injury causes overactivation of the receptor leading to accumulation of intracellular Ca^2+^, inducing apoptotic cascade cell death [6, 7]. Conversely, hypofunction of NMDA neurotransmission appears to be related to dopaminergic system dysfunction underlying the emergence of psychotic symptoms [5].

*In vivo* knowledge of NMDARs activation may provide an understanding of their implication in the physiopathology of neurodegenerative and psychiatric diseases and would help assess and develop therapeutic strategies. Several positron emission tomography (PET) tracers have been synthesized to this end [7, 8]. Most of them are NMDAR channel blockers derivatives, targeting the phencyclidine site (PCP) located inside the ion channel, thus selectively bind NMDARs in the open and active state. Unfortunately, most of these ligands have not convincingly shown a capacity to visualize opened NMDARs *in vivo*. Several families of PET ligands have been evaluated for human use but have produced inconclusive results due to non-specific binding (MK-801, phencyclidine or thienyl-phencyclidine analogs [9, 10]), rapid metabolism and washout ([^11^C]S-Ketamine [11]), or poor ability to penetrate the blood–brain barrier (BBB) (benzoquinolizinium analogs [12, 13]). More recently, diarylguanidines compounds have yielded promising results. These compounds, especially [^18^F]GE-179, are characterized by their high *in vitro* affinity for the NMDARs PCP site (*K*_*i*_ = 2.4 nM in rats for GE-179, [14]), and showed high selectivity toward other CNS receptors including glutamate ionotropic and metabotropic receptor subtypes. However, several *in vivo* studies report an important non-specific binding, a rapid plasma metabolism, and studies aiming to exhibit an *in vivo* blocking effect of channels blockers on [^18^F]-diarylguanidines binding showed discording results. These observations result from the ubiquitous distribution of NMDARs for which the proportion of opened receptors in the basal state is unknown, the confounding effect of anesthetics, and the potential existence of different binding sites inside the channel [14–18].

Fluoroethylnormemantine (FNM) is a memantine analog acting as a non-competitive NMDA receptor antagonist able to bind the open channel. The radiolabeled compound ([^18^F]-FNM) has been developed [19], and its biodistribution and safety profile have also been studied in rats [20]. Moreover, FNM recently appeared as a potent neuroprotective drug in Alzheimer’s disease [21], and its effectiveness has also been demonstrated in rodents for preventing and treating stress-related behaviors [22, 23]. It is assumed that this compound binds preferentially extra synaptic NMDARs, mostly involved in excitotoxicity, because of its chemical structure which is close to that of memantine [24, 25]. With its low molecular weight and its lipophilic properties, [^18^F]-FNM is able to cross the BBB [19]. Furthermore, its poor metabolism suggests the absence of radiometabolites likely to cross the BBB and bind to PCP site [20].

In this study, we described the *in vitro* pharmacological characterization (affinity and selectivity) of [^18^F]-fluoroethylnormemantine ([^18^F]-FNM) in rat brain and the measurement of its brain uptake in rats following stereotaxic quinolinic acid (QA) injection in the left motor area (M1). Acting as an agonist of NMDARs, QA is a neuroactive metabolite of the kynurenine pathway extensively used as an experimental model of excitotoxicity [26–28]. We hypothesized that [^18^F]FNM PET would visualize chemically-induced focal NMDARs activation as increased tracer uptake.

## Materials and Methods

### *In Vitro* Binding Assay

#### Membrane Preparation

The source of binding sites was rat brain synaptic membranes, prepared as described previously [29]. Briefly, Wistar rats (*n* = 38) were anesthetized with isoflurane and euthanatized by exsanguination. Brains were rapidly removed and the hippocampus, frontal cortex, striatum, or forebrain (brain without brainstem and cerebellum) were dissected out. Brain area fractions were homogenized in 15-fold volumes of ice-cold buffer (Tris–HCl 50 mM pH 7.5) with a Potter Elvehjem tissue grinder. The homogenates were centrifuged at 1000 × g for 10 min at 4 °C. The first supernatant was recovered and the pellets were resuspended in the same fresh buffer, homogenized, and centrifuged at 1000 × g for 10 min at 4 °C for a second time. The pellets were discarded and the two supernatants were pooled together and centrifuged at 100.000 × g for 30 min at 4 °C. The pellets were finally suspended in fresh buffer, aliquoted, and frozen at − 80 °C. Protein concentration in each fraction was determined by the Bio-Rad assay using bovine serum albumin as standard.

For α-amino-3-hydroxy-5-methyl-4-isoxazolepropionic acid (AMPA), glycine, and kaïnate receptors binding experiments, rat forebrain membranes were washed two additional times by centrifugation for 30 min at 100.000 × g at 4 °C before freezing. On the day of the experiments, rat brain membranes were thawed, incubated for 15 min at 37 °C and washed another time by resuspension in the ice-cold buffer and centrifuged at 100.000 g for 30 min at 4 °C to remove endogenous glycine and glutamate [30–32].

#### Competition Studies

Affinities of [^19^F]-FNM to the PCP site were evaluated through receptor binding assays performed with rat brain membrane preparations (hippocampus, frontal cortex, or forebrain) with [^3^H]N-(1-[thienyl]cyclohexyl)piperidine ([^3^H]TCP, 40 Ci/mmol, Perkin-Elmer) as a radioligand. Rat brain membranes (100 µg protein/tube) were incubated for 1 h at room temperature in a total volume of 100 µL of Tris–HCl buffer (50 mM, pH 7.5), including glutamate (10 µM) and glycine (10 µM) with [^3^H]TCP (10 nM) and various concentrations of [^19^F]-FNM (10^−7^–10^−3^ M) (M2I Development, LACQ France) [30]. Experiments were terminated by the addition of 5 mL of ice-cold Tris–HCl buffer 50 mM, pH 7.5 and rapid filtration under vacuum through Whatman GF/B glass fiber filters (pre-soaked in 0.3% polyethyleimine) followed by two 5 mL washes using a sampling manifold system (Millipore). Filters were transferred to vials with 4 mL of scintillation solution (EcoscintTM A, National Diagnostics) and radioactivity was measured with a Tricarb 2100TR scintillation counter (Packard). All assays were performed in triplicate. IC_50_ values (concentrations required to inhibit 50% of specific binding) were calculated by fitting displacement curves using non-linear least squares regression analysis and the inhibitory constant (*K*_*i*_) values were determined with Prism (GraphPad Sofware). The dissociation constant (*K*_*D*_) values of [^3^H]TCP in each rat brain membrane preparation used for *K*_*i*_ calculation was obtained from Vignon et al. (*K*_*D*_ = 5,5 nM in rat hippocampus, and *K*_*D*_ = 6,5 nM in rat frontal cortex), and Bénavidès et al. (*K*_*D*_ = 21 nM in rat forebrain) [31, 32].

The pharmacological selectivity profile of [^19^F]FNM was evaluated by measuring its effects on the binding of seven specific radioligands for other neurotransmitters receptors: [^3^H]glycine (45.2 Ci/mmol, Perkin-Elmer), [^3^H]AMPA (55.7 Ci/mmol, Perkin-Elmer), [^3^H]kainic acid (30 Ci/mmol, Perkin-Elmer), [^3^H]diprenorphin (25.8 Ci/mmol, Perkin-Elmer), [^3^H]DAMGO (50 Ci/mmol, Perkin-Elmer), [^3^H]SCH23390 (80 Ci/mmol, American Radiolabeled Chemicals, Inc.), and [^3^H]raclopride (80 Ci/mmol, American Radiolabeled Chemicals, Inc.). Competition binding assays were performed as described above, using several rat brain membrane preparations. Experimental protocols were first validated using homologous competition and are documented in Table 1.

**Table 1:** Experimental protocols for competition binding assays

Putative receptor	Radioligand	Buffer	Rats brain membranes	Incubation condition	Reference
NMDA, glycine site	[^3^H]glycine (50 nM)	Tris–HCl (50 mM, pH 7,5) containing strychnine (100 µM)	Rat forebrain (100 µg protein)	60 min; 4 °C	[33]
AMPA receptor	[^3^H]AMPA (10 nM)	Tris–HCl (50 mM, pH 7,5) containing KSCN (100 mM)	Rat forebrain (100 µg protein)	60 min; 4 °C	[34]
Kaïnic acid receptor	[^3^H]kaïnic acid (20 nM)	Tris–HCl (50 mM, pH 7,5)	Rat forebrain (100 µg protein)	120 min; 4 °C	[35]
Opioid receptor	[^3^H]diprenorphine (1 nM)	Tris–HCl (50 mM, pH 7,5)	Rat forebrain (50 µg protein)	60 min; room temperature	[36]
µ-opioid receptor	[^3^H]DAMGO (1 nM)	Tris–HCl (50 mM, pH 7,5)	Rat forebrain (50 µg protein)	60 min; room temperature	[36]
Dopaminergic receptor (D_1_)	[^3^H]SCH23390 (1 nM)	Tris–HCl (50 mM, pH 7,5) containing NaCl (120 mM), MgCl_2_ (1 mM)	Rat striatum (50 µg protein)	60 min; room temperature	[37]
Dopaminergic receptor (D_2_)	[^3^H]raclopride (1 nM)	Tris–HCl (50 mM, pH 7,5) containing NaCl (120 mM), MgCl_2_ (1 mM)	Rat striatum (50 µg protein)	60 min; room temperature	[38]

### *In Vivo* Study

#### Animals

This study was conducted under protocols approved by the French Animal Ethics Committee (No. 2016021711398144). Sixteen Sprague Dawley female rats were used for this study (300–350 g, over 11 weeks old, Elevage Janvier, LeGenest-St-Isle, France). The animals were housed in a climate-controlled room with a 12/12-h light cycle and fed standard rat chow and water ad libitum. During housing, animals were monitored twice a day for health status over 24 h post-surgery and then daily.

#### Experimental Design

We randomized 16 rats into two groups: 9 control rats without lesion and 7 injured rats with stereotaxic QA injection in the left hemisphere. To minimize the number of animals used, 3 injured rats also received stereotaxic injection of 0.9% saline solution (NaCl) in the right hemisphere in order to evaluate the effect of stereotaxic injection on [^18^F]FNM fixation. Control rats were imaged once while lesioned rats were imaged 24 and 72 h after injury.

#### Stereotaxic Lesions

Seven adult female Sprague–Dawley rats were anesthetized with pentobarbital (50 mg/kg, intraperitoneal injection) and premedicated with methylprednisolone 0.4 mg/kg. Cortical lesions focused on the caudal forelimb motor area (M1) were induced by QA stereotaxic injection into the left hemisphere at the following stereotaxic coordinates: antero-posterior 0, 2.5 mm lateral to Bregma, and 2 mm deep [39]. QA, an agonist of NMDARs acting as an excitotoxin, was injected at a rate of 0.4 μL/min using a Hamilton micro syringe fitted with a 22G needle. After the infusion of 2 μl (300 nM), the cannula was left in place for 5 min to allow complete diffusion of the injected solution. The incision was sutured and the rats were allowed to recover in their normal environment.

#### Radiotracer Preparation

[^18^F]FNM was synthetized according to a previously described method [19]. Briefly, [^18^F]FNM was produced by nucleophilic substitution using 1-[N-(tert-butyloxy)carbamoyl]-3-(tosyl)ethyl-adamantane as the precursor on a Raytest® module. After complete removal of the solvent by azeotropic drying, the precursor was added to the reaction vial and was heated for 20 min at 125 °C. The reaction mixture was cooled, added to the hydrolysis solution (hydrochloric acid 6 N) and heated for 10 min at 110 °C, causing hydrolysis of the BOC (tert-butoxycarbonyle) group. The reaction mixture was then neutralized by adding 6 N NaOH and 0.5 M trisodium citrate solution. Pre-purification was achieved using a Sep-Pak cartridge (waters C18 Plus). The lipophilic compound trapped in the cartridge was eluted with 2 mL of ethanol. HPLC purification was carried out on a semi-preparative column (Cluzeau Info Labo Stability Basic C-18 CIL; 250 × 10 mm, particle size 5 μm) with a mobile phase consisting of 100% ethanol/sodium acetate (0.1 M) mixture (45/55, v/v). The [^18^F]FNM retention time was 15 min with a flow rate of 2 mL/min. Post-purification was performed in order to obtain a solution containing 10% of ethanol and 90% of saline.

#### Image Acquisition

Imaging was performed with a hybrid microPET/CT (NanoScan/CT, Mediso), 24 and 72 h after injury. Animals were initially anesthetized with an isoflurane mixture (4% induction, 2% maintenance, carrier gas medical air/O_2_ 50/50 0.5 L/min). Acquisition was performed 40 min after injection of 30 MBq + / − 20 of [^18^F]-FNM in a heparinized catheter in the tail vein. CT (computed tomography) acquisitions were performed during 3.5 min (parameters: 50 kVp, 800 μA) followed by whole-body static PET images (energy window: 400–600 keV) in list mode during 30 min and reconstructed using 3D mode (Tetra-Tomo3D Mediso) with 4 iterations and 6 subsets. The size of the images initially reconstructed (mm) was 470 × 388 × 750 (x,y,z). The voxel size (μm) was 126 × 126 × 126. All images were automatically corrected for radioactive decay due to manufacturer software settings. Following the reconstruction, the CT images were spatially aligned to match the PET images. In addition to image reconstruction, the CT data were used for attenuation correction of PET images.

#### Image Processing, Co-Registration, and Statistical Analysis

**Fig. 1.**
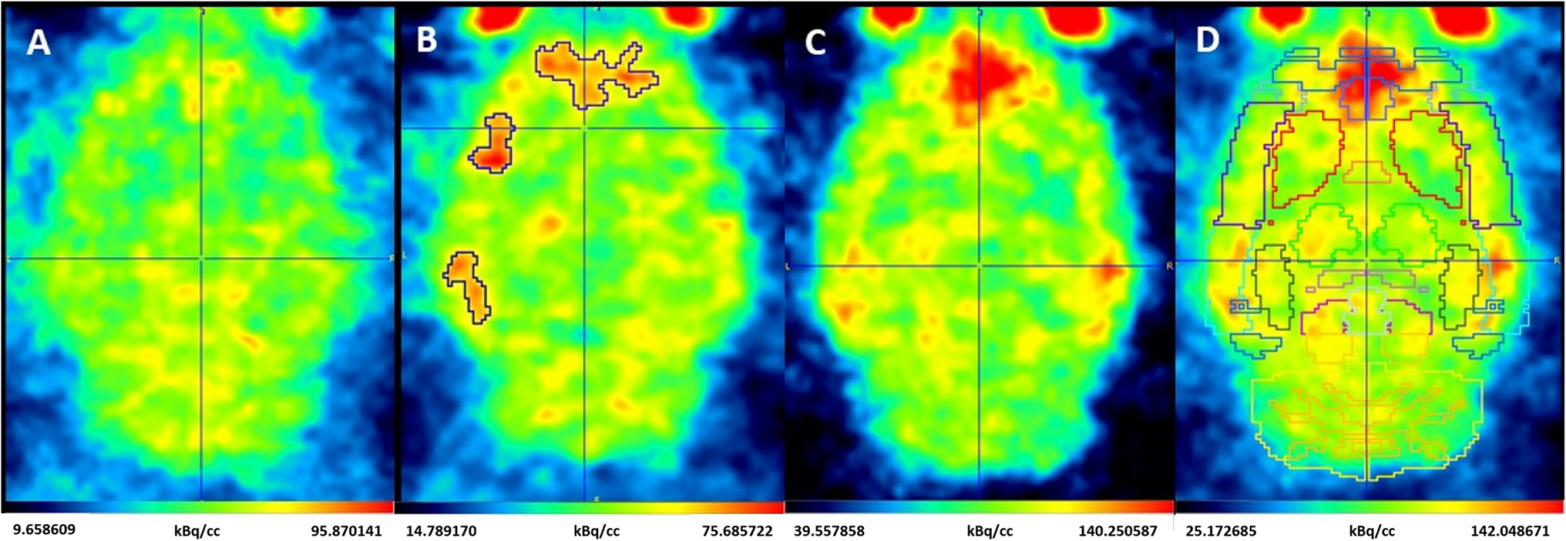
[^18^F]-FNM PET images of control rat (**A**), injured rat 24 h (**B**), and 72 h after injury (**C** and **D**, with Rat_W.Schiffer atlas). Imaged from injured rats 24 h and 72 h after injury (**B**, **C**, and **D**) are not the same subject.

We have performed three types of data analysis:

1. Z-score analysis: The nine cerebral images of rats without lesion were realigned and underwent an elastic registration allowing the creation of a template (FSL software). Then, a descriptive voxel-wise analysis with SPM software was carried out in order to determine a Z-score map between injured and controls rats. Z-score = [(individual value)—(control mean)]/(control SD). With MRICron software, Z-score map between each injured rat and control rats template obtained 24 and 72 h after injury were co-registered with elastic transformations to an average rat brain atlas (Rat W. Schiffer, [40]). Left and right cortex were then delineated on each Z-score map. Anatomical regions of interest were classified as follows: *QA_left_cortex*, *no_NaCl_right_cortex* and *NaCl_right_cortex*. Then, the percentage rate of voxels with a Z-score value exceeding the threshold value of 3 was determine in each anatomical region in order to highlight differences of [^18^F]FNM uptake with QA or NaCl stereotaxic injection.
2. SUVR of each brain area after brain segmentation: With PMOD Software (v3.905), the rat W. Schiffer brain atlas was applied on injured and control rats (Fig. 1) in order to delineate volumes of interest (VOI) corresponding to brain areas. The standard uptake values (SUV) of each VOI were computed. In order to standardize data, a ratio of brain areas to that in the whole cerebellum was calculated (SUVR). The homogeneity of cerebellar SUVs between groups was tested using one-way ANOVA. The difference in [^18^F]FNM uptake in control and injured rats 24 or 72 h after injury in each cortical area was then interrogated with an unpaired one-tailed Student *t*-test applied on the mean SUVR between each group. In order to verify the absence of [^18^F]FNM uptake in the right hemisphere of rats having received saline stereotaxic injection, SUVR of the NaCl injection area has also been compared between groups using one-way ANOVA. For each analysis, a Fisher test was used to assess variance homogeneity. In case of non-homogeneous variances, Welch *t*-test have been performed.
3. Manual VOI delineation of [^18^F]FNM uptake on injured rats: QA having diffused in a variable way in cortical areas according to the individuals, we visually delineated increased [^18^F]FNM uptake areas in each injured rat. SUVRs of these areas were then compared with the corresponding areas in the control group using the unpaired one-tailed Student *t*-test.

Normal distribution of data was confirmed using the Shapiro–Wilk test. In the case of a non-Gaussian data distribution, a Mann–Whitney test was performed instead of the Student test. All statistical analysis was performed using GraphPad Prism Software (v9.3.1). Where applicable, results are presented as mean ± standard deviation (SD).

## Results

### *In Vitro* Binding Assays

**Fig. 2.**
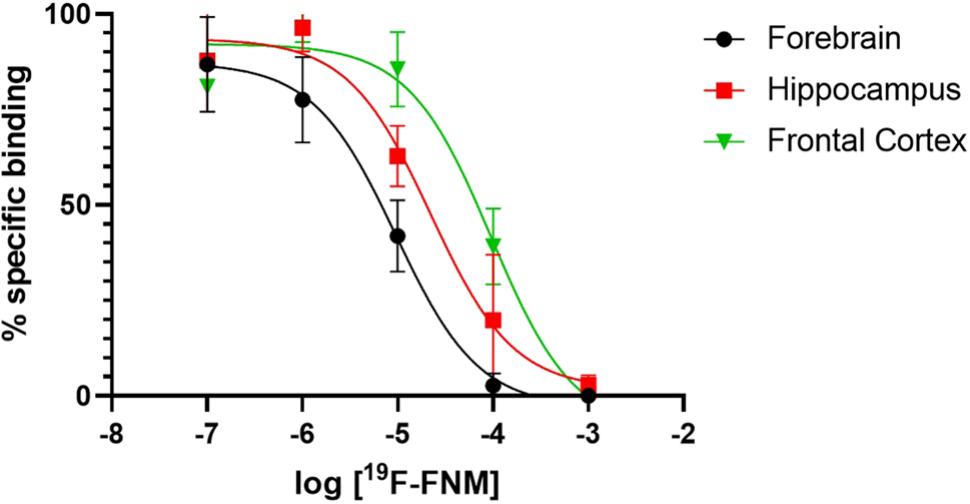
Heterologous inhibition curves. Inhibition of the binding of [^3^H]TCP by increasing concentrations of [^19^F]FNM in several rat brain membrane suspensions. Percentage of non-specific binding: 69.57 ± 4.78% in forebrain, 50.06 ± 9.22% in hippocampus, and 51.55 ± 4.85% in frontal cortex.

The affinity of [^19^F]FNM for the NMDA PCP site was measured by the inhibition of [^3^H]TCP binding in different rat brain membrane suspensions. Results appear in Table 2 and Fig. 2. IC_50_ values are 13 ± 9 µM in the forebrain, 80.8 ± 15.1 µM in the frontal cortex, and 52.9 ± 9.7 µM in the hippocampus.

**Table 2:** Binding affinity of [^19^F]FNM to the NMDA PCP site

Rat brain membranes suspensions	IC_50_^a^ ± SEM (M)	*K*_*i*_^b^ ± SEM (M)
Frontal cortex	8.08 ± 1.51.10^−5^	3.39 ± 0.72.10^−5^
Hippocampus	5.29 ± 0.97.10^−5^	1.92 ± 0.14.10^−5^
Forebrain	1.30 ± 0.89.10^−5^	7.81 ± 5.36.10^−6^

a: IC_50_ is the concentration of [^19^F]FNM inhibiting 50% of [^3^H]TCP binding; b: *K*_*i*_ was obtained by transforming IC_50_ values according to the Cheng and Prusoff equation. For the transformation, K_D_ values of the [^3^H]TCP in hippocampus, frontal cortex and forebrain was obtained from the literature [31, 32]

As shown by the results summarized in Table 3, [^19^F]FNM did not displace specific radioligands of the glycine site, AMPA, kainite, or dopaminergic receptors. It is able to bind opioid receptors with a low affinity.

**Table 3:** Affinity of [^19^F]FNM for other CNS receptors

Target	Radioligand	Ligand used as positive control	IC_50_ ± SEM (Homologous inhibition/Positive control) (M)	IC_50_ ± SEM ([^19^F]FNM inhibition) (M)
NMDA receptor glycine site	[^3^H]-glycine	L-glycine	3.5 ± 2.2.10^−7^	No inhibition
AMPA receptor	[^3^H]-AMPA	AMPA	2.7 ± 1.7.10^−7^	No inhibition
Kaïnate receptor	[^3^H]-kainic acid	Kaïnic acid	6.5 ± 2.5.10^−8^	No inhibition
Opioïd receptors	[^3^H]-diprenorphine	Diprenorphine	5.4 ± 2.4.10^−10^	9.6 ± 2.8 .10^−5^
µ opioïd receptor	[^3^H]-DAMGO	DAMGO	9.1 ± 0.8.10^−10^	1.4 ± 0.2.10^−4^
Dopaminergic receptor (D1)	[^3^H]-SCH23390	SCH23390	6.2 ± 2.4.10^−9^	> 10^−3^
Dopaminergic receptor (D2)	[^3^H]-raclopride	Butaclamol	7.6 ± 3.6.10^−9^	> 10^−3^

### *In vivo* study

#### Z-Score Analysis

**Fig. 3.**
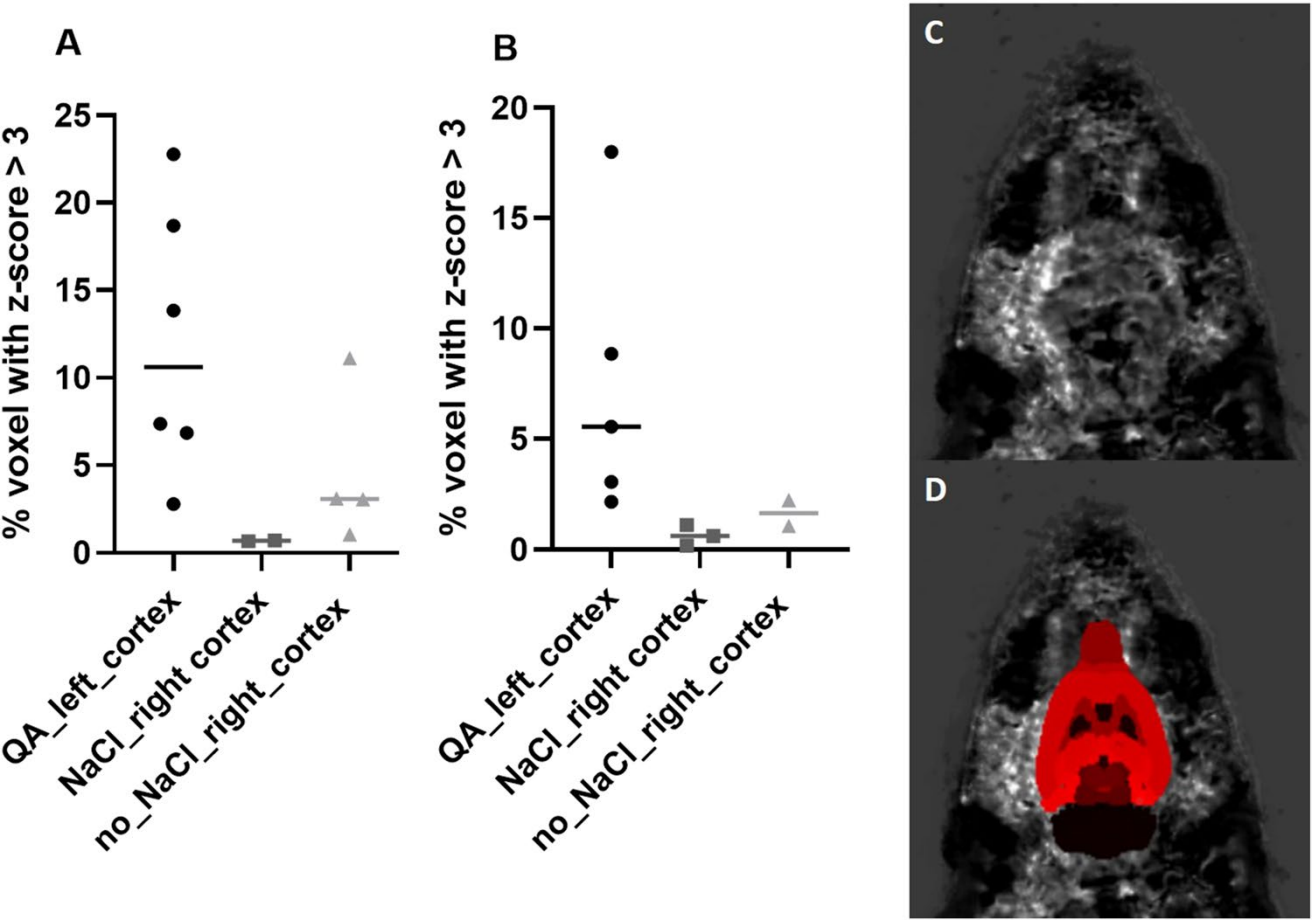
**A**, **B** Percentage of voxels with a Z-score value exceeding the threshold value of 3 in left cortex of injured rats, and right cortex of injured rats with or without NaCl stereotaxic injection, 24 h (**A**) or 72 h (**B**) after injury. **C**, **D** Z-score map without (**C**) and with (**D**) rat brain Schiffer atlas.

Twenty-four and seventy-two hours after stereotaxic QA injection, 12.1 ± 7.7% and 7.5 ± 6.4% of the voxels of the left cortex presented a Z-score exceeding a threshold of Z-score > 3 and therefore a [^18^F]FNM uptake significantly higher than controls (Fig. 3 and Table 4). In the right cortex, the percentage of voxels with a Z-score higher than 3 were 0.7 ± 0.03% and 4.6 ± 4.5% with and without NaCl stereotaxic injection, respectively, 24 h after injury and 0.7 ± 0.5% and 1.7 ± 0.8% 72 h post-injury. Stereotaxic injection alone did not affect [^18^F]FNM uptake. Unlike the left cortex, there were very few significant voxels in the right cortex with and without NaCl stereotaxic injection.

**Table 4:** Mean number of voxels ± SD with z-score > 3 in rats brains cortex

	24 h post-injury		72 h post-injury	
*Data*	Number of voxel	%	Number of voxel	%
*QA_left_cortex*	2630 ± 686	12.06 ± 7.71	1644 ± 624	7.54 ± 6.40
*no_NaCl_right_cortex*	1001 ± 286	4.59 ± 4.46	361 ± 126	1.66 ± 0.82
*NaCl_right cortex*	153 ± 4	0.70 ± 0.03	141 ± 58	0.65 ± 0.46

#### SUVR of Each Brain Area After Brain Segmentation

**Fig. 4.**
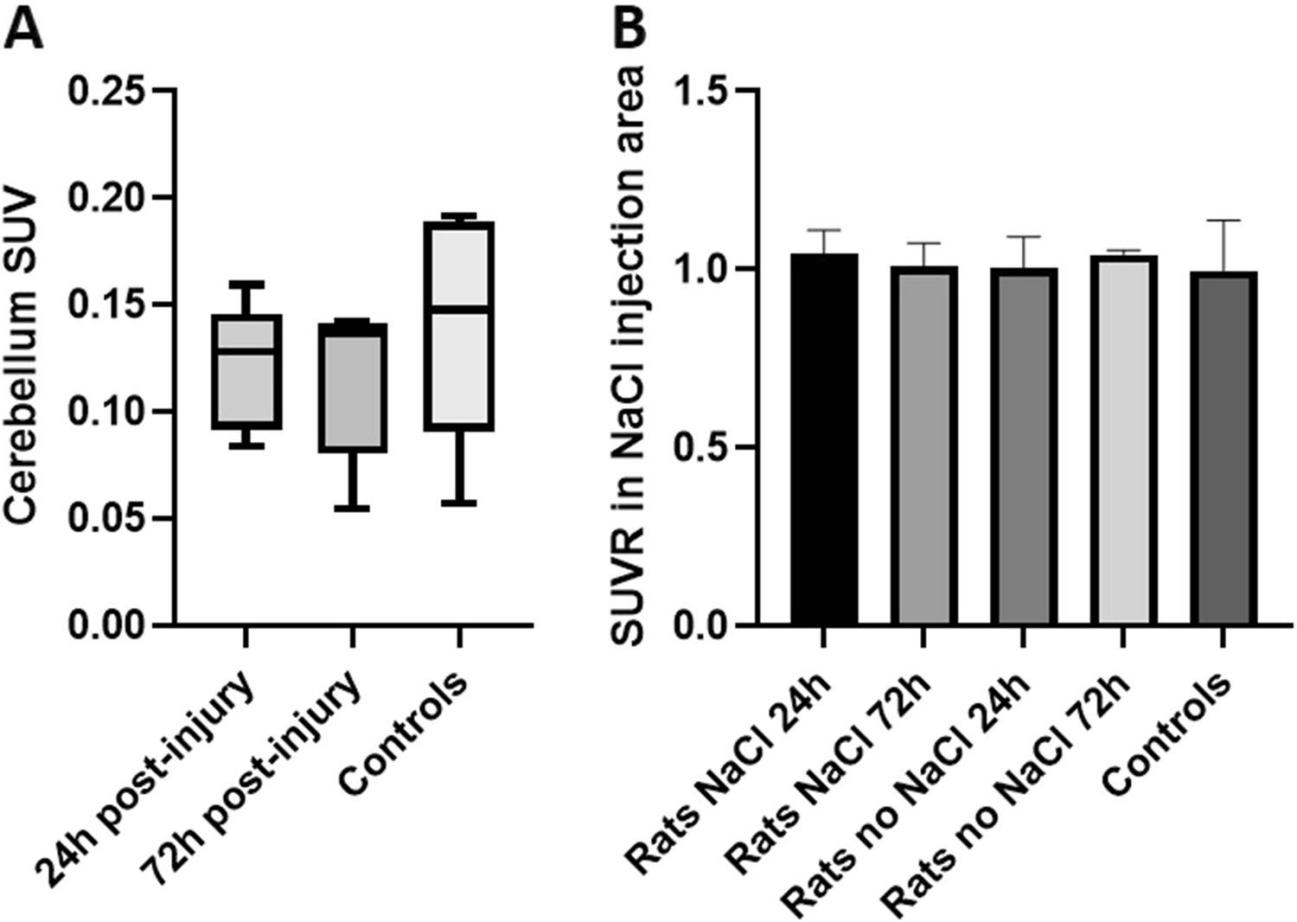
**A** Mean cerebellum SUV ± SD of [^18^F]FNM uptake in cerebellum. **B** Mean SUVRs ± SD of [^18^F]FNM uptake in NaCl injection area in controls and injured rats with and without NaCl stereotaxic injection 24 h and 72 h after injury. SUVR = SUV NaCl injection area/SUV cerebellum.

There was no significant difference between mean cerebellum SUVs (one-way ANOVA, *p* value = 0.54) in all groups, see Fig. 4A. The cerebellum was, therefore, taken as a reference to calculate the SUVRs of each cortical area delimited using the Schiffer atlas. Accordingly, to z-score analysis, we did not observe significant difference of [^18^F]FNM uptake in the NaCl injection area between groups, see Fig. 4B (one-way ANOVA, *p* value = 0.96).

**Fig. 5.**
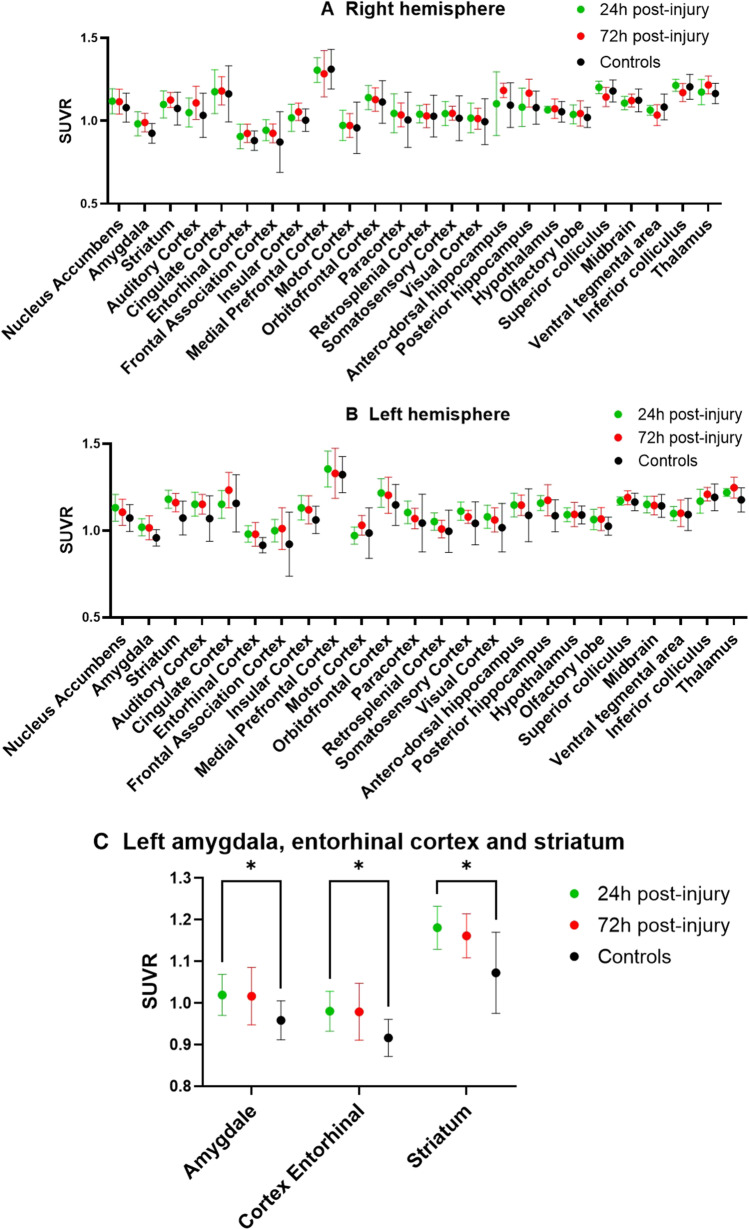
Mean SUVRs ± SD of [^18^F]FNM uptake. SUVR = SUV area/SUV cerebellum in right (**A**) and left hemispheres (**B**) in injured rats and controls. **C** Left hemisphere areas in which [^18^F]FNM uptake is significantly different between injured and controls.

There is a significant difference of [^18^F]FNM uptake in the left hemisphere in the entorhinal cortical area (*p* value = 0.015), striatum (*p* value = 0.019), and amygdala (*p* value = 0.024) between injured rats 24 h after injury and controls (Fig. 5). These differences are no longer significant after 72 h. No significant difference was observed between the groups in the brain areas of the right hemisphere 24 h and 72 h after injury.

#### Manual VOI Delineation of [^18^F]FNM Uptake on Injured Rats

**Fig. 6.**
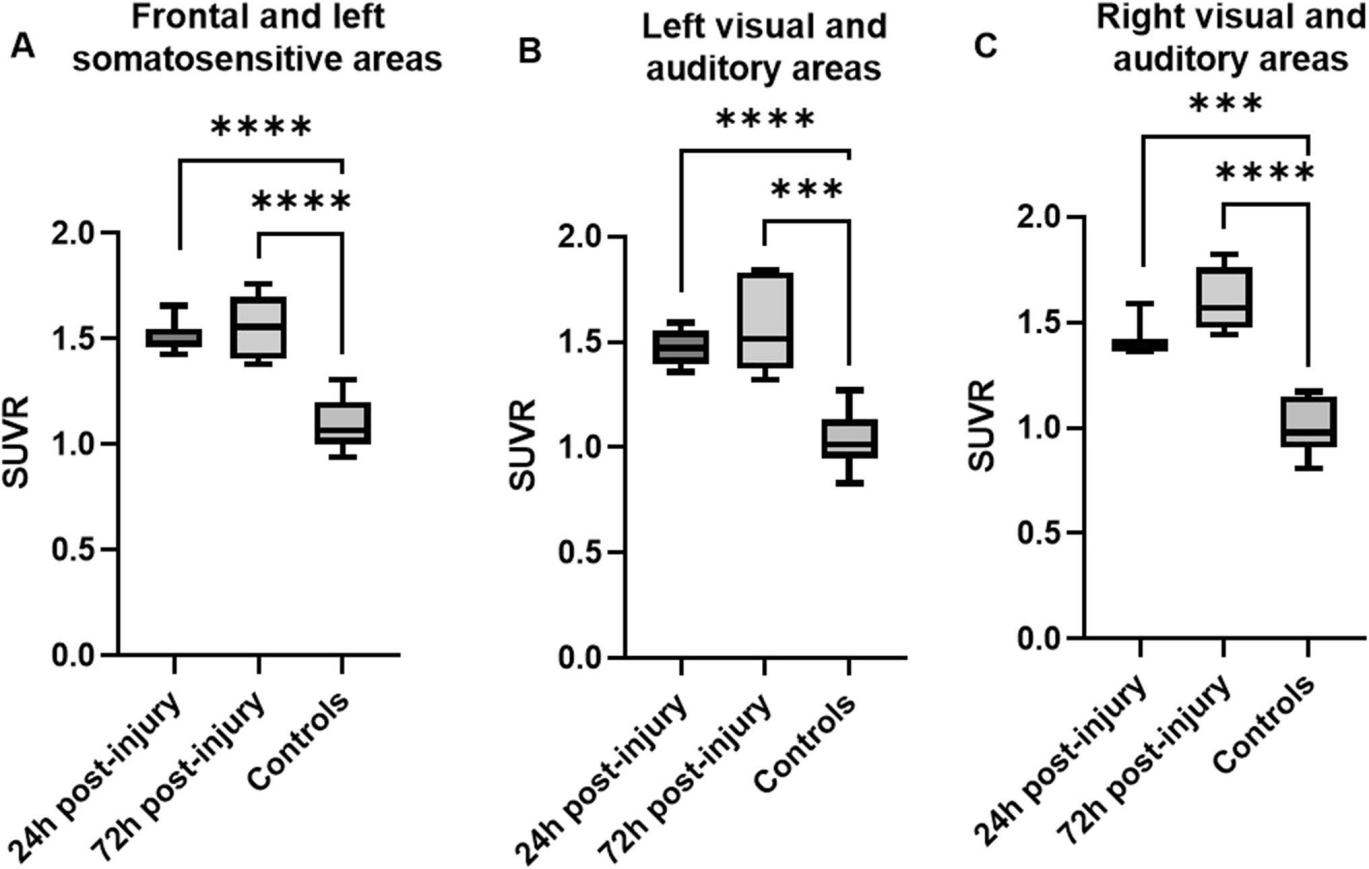
Mean SUVR ± SD of [^18^F]FNM uptake in manually delineated VOIs in injured rats compared to those of the same areas in controls. [.^18^F]FNM uptake increased in 3 cortical regions depending on the individuals: frontal and left somatosensory areas (**A** *n* = 7, VOI volume = 3.06 ± 1.70.10–2 ccm, and *n* = 5, VOI volume = 2.13 ± 2.26.10–2 ccm, 24 h and 72 h post-injury), left visual and auditory areas (**B** *n* = 6 VOI volume = 6.25 ± 3.84.10–3 ccm, and *n* = 5 VOI volume = 4.38 ± 4.08.10–3 ccm 24 h and 72 h post-injury), and right visual and auditory areas (**C** *n* = 3 VOI volume = 2.97 ± 2.39.10–3 ccm, and *n* = 4 VOI volume = 5.28 ± 4.24.10–3 ccm 24 h and 72 h post-injury).

In the frontal and left somatosensory areas, [^18^F]FNM PET detected a mean of 37% and 41% increase in [^18^F]FNM uptake (*p* < 0.0001) 24 and 72 h, respectively, after QA stereotaxic injection compared to the control group, see Fig. 6A. The mean SUVR of the [^18^F]FNM uptake VOI was 1.51 ± 0.08 and 1.55 ± 0.15 24 h and 72 h after injury compared to 1.10 ± 0.12 in the frontal and somatosensory areas of controls. [^18^F]FNM uptake was also found in some injured rats in the left and right visual and auditory areas (Fig. 6B and C). Uptake was significantly higher in injured rats 24 h (*p* < 0.0001 on the left side and *p* = 0.0006 on the right side) and 72 h (*p* = 0.0001 on the left side and *p* < 0.0001 on the right side) after the lesion compared to controls.

## Discussion

The aim of this study was to evaluate the ability of [^18^F]-FNM to localize activated NMDA receptors in a rat preclinical model of excitotoxicity. *In vitro* binding experiments were carried out to determine the affinity of [^19^F]-FNM for the PCP site of NMDARs and other CNS receptors. As shown in Table 2, [^19^F]-FNM inhibited the binding of the NMDA-type receptor antagonist [^3^H]-TCP with different IC_50_ values according to the membranes used. These different affinities can be explained by the variability of receptor subunit composition in different areas. This range of values is similar to those reported by Salabert et al. [19] and confirms that [^18^F]-FNM belongs to the class of moderate to low affinity uncompetitive antagonists of NMDARs such as memantine or [^18^F]-MEM [41, 42]. We also examined the selectivity profile of [^19^F]-FNM with respect to various other cerebral receptors. These targets were chosen from a previous study on the *in vitro* evaluation of another molecule having a similar chemical structure to FNM, 1-amino-3-[^18^F]fluoromethyl-5-methyl-adamantane ([^18^F]-MEM) [41]. In this study, *in vitro* receptor screening was performed to measure the inhibitory effects of [^19^F]-MEM on different CNS receptors. We selected those for which the percentage inhibition (PI) of [^19^F]-MEM was greater than 10% and therefore on which FNM was most likely to bind. Among these targets, [^19^F]-FNM did not displace specific radioligands of the glycine site, AMPA, kainate, and dopaminergic receptors. It is able to bind opioids but, in the forebrain, the selectivity of FNM for the NMDA receptor ion-channel was at least sevenfold higher compared to the other targets examined. Opioid binding could cause PET images analysis difficulties. With FNM’s IC_50_ value towards NMDAR PCP site being in the same order of magnitude as the concentration found *in vivo* in the brain after PET tracer injection, it can be assumed that *in vivo* FNM uptake is due to the binding of the tracer on opened NMDARs.

We decided to perform our study of *in vivo* imaging NMDAR activation with [^18^F]FNM PET using QA stereotaxic injection. Stereotaxic injection of the naturally occurring NMDA agonist QA serves as a valuable *in vivo* model to study excitotoxic cell damage in the CNS [28, 43, 44]. Our aim was to open NMDARs on a large scale in order to visualize if [^18^F]FNM PET could detect this activation before testing this imaging technique on another preclinical model in which the receptors would be activated on the smallest well-delineated zone. For example, the hippocampus could be an interesting target for further studies, due to the dysfunction of NMDARs found in this area in various pathologies such as schizophrenia [45] or Alzheimer’s disease [46]. According to the literature, QA has no influence on the bioavailability of opioid receptors [28, 47, 48], therefore, even if FNM can bind to it with a low affinity, the uptake is indeed due to an opening of the NMDARs. Moreover, the z-score analysis and comparison of SUVRs in the NaCl injection area between controls and injured rats shows that saline stereotaxic injection does not cause NMDAR opening. In the right cortex, the percentage of voxels with a Z-score exceeding 3 was slightly higher with saline stereotaxic injection, see Fig. 3A. This may be the result of changes in the QA dissemination induced by NaCl injection. Thus, injured rats with or without NaCl injection in the right motor areas were not treated separately in subsequent analyses. With mean cerebellum SUVs being not different between groups (see Fig. 4A), we chose to use the whole cerebellum as a reference region for SUVR calculation.

SUVRs analysis after brain segmentation on PMOD using Schiffer’s atlas [40] highlights a significantly greater uptake (*p* < 0.05) of [^18^F]FNM in the left striatum, entorhinal cortex, and amygdala of injured rats 24 h after AQ injection, compared with control rats. These differences were not observed in the right hemisphere, which suggests that they are due to NMDARs opening in these regions following QA injection. However, no significant differences are observed between injured rats and controls, 72 h after injury. Although the amount of QA injected and the positioning of the injection needle was the same in all rats, QA diffusion through the brain was not homogenous and varied according to the individuals. [^18^F]FNM uptake areas straddled the borders of different brain regions in varying volumes. Therefore, it is very difficult to highlight these areas by comparing the average SUVRs of each brain region. This approach is therefore not suitable to objectify differences in [^18^F]FNM binding which may be due to preclinical model variability.

One limitation of this study was the low number of rats in each group, especially 72 h after injury because two rats died in the time interval between the two imaging phases which reduced our power to detect QA-induced NMDAR activation. This preclinical model using QA stereotaxic injection resulted in various effect sizes of [^18^F]FNM uptake that were nonetheless highly significant despite the low number of rats. However, this study shows that QA lesions are very difficult to delineate because of the non-reproducibility of QA diffusion. This leads to an unequal distribution of SUVR values in tracer-binding areas but also to a great variability of volume in these areas. We therefore had no choice but to delineate these regions manually.

The third analysis, with manually delineated VOI, showed that [^18^F]FNM PET can detect *in vivo* activated NMDARs following QA stereotaxic injection in rats. [^18^F]FNM uptake is mostly seen in the frontal area (including cingulate, frontal association, medial prefrontal, and orbitofrontal cortex) and in the left somatosensory cortex. There is no [^18^F]FNM uptake in the motor cortex where QA is injected, possibly due to widespread brain tissue damage in this area. The bevel of the needle pointing forward during the injection means QA diffusion is directed towards frontal and somatosensory areas. [^18^F]FNM uptake is also observed in some rats in the right and left visual and auditory areas. This phenomenon may be due to the existence of neural networks between these two regions as described by Boonzaier et al. [49].

## Conclusions

In spite of its moderate affinity and selectivity toward NMDAR, [^18^F]FNM PET imaging makes it possible to visualize major excitotoxicity in the rat brain. Indeed, in our *in vivo* study, [^18^F]-FNM uptake allowed us to delineate areas of excitotoxicity as injured areas but also other areas with secondary NMDAR activation. This promising result provides the means to envisage the use of this tracer in order to study pathologies in which abnormal activity of NMDAR is suspected. We also showed that we could use a homogeneous reference zone, namely the cerebellum, to help with image analysis. NMDAR activation is one of the most complicated imaging targets because of its variability and the difficulty of tracer access to the binding site. *In vivo* visualization of its activation is therefore especially challenging.
